# Critical Involvement of the ATM-Dependent DNA Damage Response in the Apoptotic Demise of HIV-1-Elicited Syncytia

**DOI:** 10.1371/journal.pone.0002458

**Published:** 2008-06-18

**Authors:** Jean-Luc Perfettini, Roberta Nardacci, Mehdi Bourouba, Frédéric Subra, Laurent Gros, Claire Séror, Gwenola Manic, Filippo Rosselli, Alessandra Amendola, Peggy Masdehors, Luciana Chessa, Giuseppe Novelli, David M. Ojcius, Jan Konrad Siwicki, Magdalena Chechlinska, Christian Auclair, José R. Regueiro, Hugues de Thé, Marie-Lise Gougeon, Mauro Piacentini, Guido Kroemer

**Affiliations:** 1 INSERM U848, Institut Gustave Roussy, Villejuif, France; 2 National Institute for Infectious Diseases “Lazzaro Spallanzani”, Rome, Italy; 3 CNRS UMR 8113 LBPA, Ecole Normale Supérieure de Cachan, Cachan, France; 4 CNRS FRE2939, Institut Gustave Roussy, Villejuif, France; 5 Antiviral Immunity, Biotherapy and Vaccine Unit, Department of Infection and Epidemiology, Institut Pasteur, Paris, France; 6 II Faculty of Medicine, University of Rome “La Sapienza”, Rome, Italy; 7 Department of Biopathology and Diagnosing Imaging, University of Rome “Tor Vergata”, Rome, Italy; 8 School of Natural Sciences, University of California Merced, Merced, California, United States of America; 9 Department of Immunology, M. Sklodowska-Curie Memorial Cancer Center and Institute of Oncology, Warsaw, Poland; 10 Imunología, Facultad de Medicina, Universidad Complutense, Madrid, Spain; 11 CNRS UPR 9051, Université de Paris VII, Hôpital St. Louis, Paris, France; 12 Department of Biology, University of Rome “Tor Vergata”, Rome, Italy; National Institutes of Health, United States of America

## Abstract

DNA damage can activate the oncosuppressor protein ataxia telangiectasia mutated (ATM), which phosphorylates the histone H2AX within characteristic DNA damage foci. Here, we show that ATM undergoes an activating phosphorylation in syncytia elicited by the envelope glycoprotein complex (Env) of human immunodeficiency virus-1 (HIV-1) in vitro. This was accompanied by aggregation of ATM in discrete nuclear foci that also contained phospho-histone H2AX. DNA damage foci containing phosphorylated ATM and H2AX were detectable in syncytia present in the brain or lymph nodes from patients with HIV-1 infection, as well as in a fraction of blood leukocytes, correlating with viral status. Knockdown of ATM or of its obligate activating factor NBS1 (Nijmegen breakage syndrome 1 protein), as well as pharmacological inhibition of ATM with KU-55933, inhibited H2AX phosphorylation and prevented Env-elicited syncytia from undergoing apoptosis. ATM was found indispensable for the activation of MAP kinase p38, which catalyzes the activating phosphorylation of p53 on serine 46, thereby causing p53 dependent apoptosis. Both wild type HIV-1 and an HIV-1 mutant lacking integrase activity induced syncytial apoptosis, which could be suppressed by inhibiting ATM. HIV-1-infected T lymphoblasts from patients with inactivating ATM or NBS1 mutations also exhibited reduced syncytial apoptosis. Altogether these results indicate that apoptosis induced by a fusogenic HIV-1 Env follows a pro-apoptotic pathway involving the sequential activation of ATM, p38MAPK and p53.

## Introduction

In spite of the spectacular success of highly active anti-retroviral therapy (HAART), HIV-1 infection continues to pose a public health problem because an ever increasing percentage of viral strains have acquired resistance against the major anti-retroviral drugs [1]. Although HAART and antibiotic therapy have reduced the frequency of opportunistic infections, they have been less successful in preventing HIV-1-linked neoplastic diseases and the HIV-1-associated encephalopathy (HAE), which may lead to dementia [2], [3].

One of the major pathogenic mechanisms of the acquired immunodeficiency syndrome (AIDS) is the apoptotic destruction of HIV-1-infected cells (“direct killing”) as well as the death of non-infected cells, many of which are immunologically relevant (“bystander killing”) [4]–[6]. HIV-1 encodes for several apoptogenic proteins including Env, Vpr and Tat [4]–[7]. Among these proteins, Env is particularly important, because viruses in which Env has been replaced by the envelope of vesicular stomatitis virus (VSV) lose much of their apoptogenic activity [8], [9]. The membrane-anchored Env gp120/gp41 complex expressed on the surface of HIV-1-infected cells can induce apoptosis through an interaction with uninfected cells expressing the receptor (CD4) and the chemokine co-receptor (CXCR4 or CCR5). This type of bystander killing often involves the formation of syncytia by fusion of the interacting cells. Syncytium formation (cytogamy) leads to apoptosis [10], and this mode of cell death induction participates in the AIDS-associated depletion of immune cells. Thus, a positive correlation between CD4^+^ T cell decline and infection by syncytium-inducing HIV-1 or SIV-1 variants has been established in vitro [11] and, more importantly, in vivo, in monkeys [12], humanized SCID mice [13] and humans with AIDS [14], [15]. Syncytia or “multinuclear giant cells” accumulating in the cerebral cortex are pathognonomic for HAE [16], [17], meaning that their presence allows for the histopathological diagnosis of neuro-AIDS.

Syncytia elicited by HIV-1 succumb to apoptosis following a complex pathway involving the activation of several kinases (cyclin-dependent kinase-1, Cdk1; mammalian target of rapamycin, mTOR; p38 mitogen-activated protein kinase, p38 MAPK; inhibitor of NF-κB kinase, IKK), as well as the activation of several transcription factors (NF-κB, p53), finally resulting into the activation of the mitochondrial pathway of apoptosis [18]. p53 is the major pro-apoptotic transcription factor involved in this pathway because its inhibition suppresses cell death and normalizes ∼80% of the genes that are differentially up- or downregulated upon syncytium formation [19]. Activation of p53 can be detected by the use of phospho-neoepitope-specific antibodies that recognize p53 phosphorylated on serine 15 (p53S15P) or serine 46 (p53S46P). Indeed, syncytia elicited by HIV-1 Env in vitro as well as multinuclear giant cells found in HAE brains are p53S15P^+^ and p53S46P^+^ [20], [21]. Overall p53 levels are increased in the brains of patients with HAE [22], [23]. Moreover, p53S15P^+^ and p53S46P^+^ cells can be detected in lymphoid organs from HIV-1 infected individuals, correlating with viral load and the treatment status [19], [20], [24]. We have identified the principal kinases responsible for p53 phosphorylation, namely mTOR (for S15) [24], [25] and p38MAPK (for both S15 and S46) [20]. Inhibition of p38MAPK completely abolishes syncytial apoptosis, and activated p38MAPK have been encountered in the same cells that contain p53S15P^+^ and p53S46P^+^ nuclei, in vitro, ex vivo and in vivo [20], [21].

Based on these premises, we decided to characterize the upstream events leading to p38MAPK and p53 activation. Here, we report that a potent tumor suppressor protein, ataxia telangiectasia mutated (ATM), which previously has been implicated in the control of p53 in tumors [26], [27], plays a major role in p53 activation in HIV-1 infection both in vitro and in vivo. We also provide evidence that activated ATM aggregates together with its substrate H2AX in so-called DNA damage foci [28]–[30] within the nuclei from syncytia elicited by HIV-1 in vitro and in vivo.

## Results

### Pre-apoptotic activation of ATM in karyogamic syncytia

**Figure 1:**
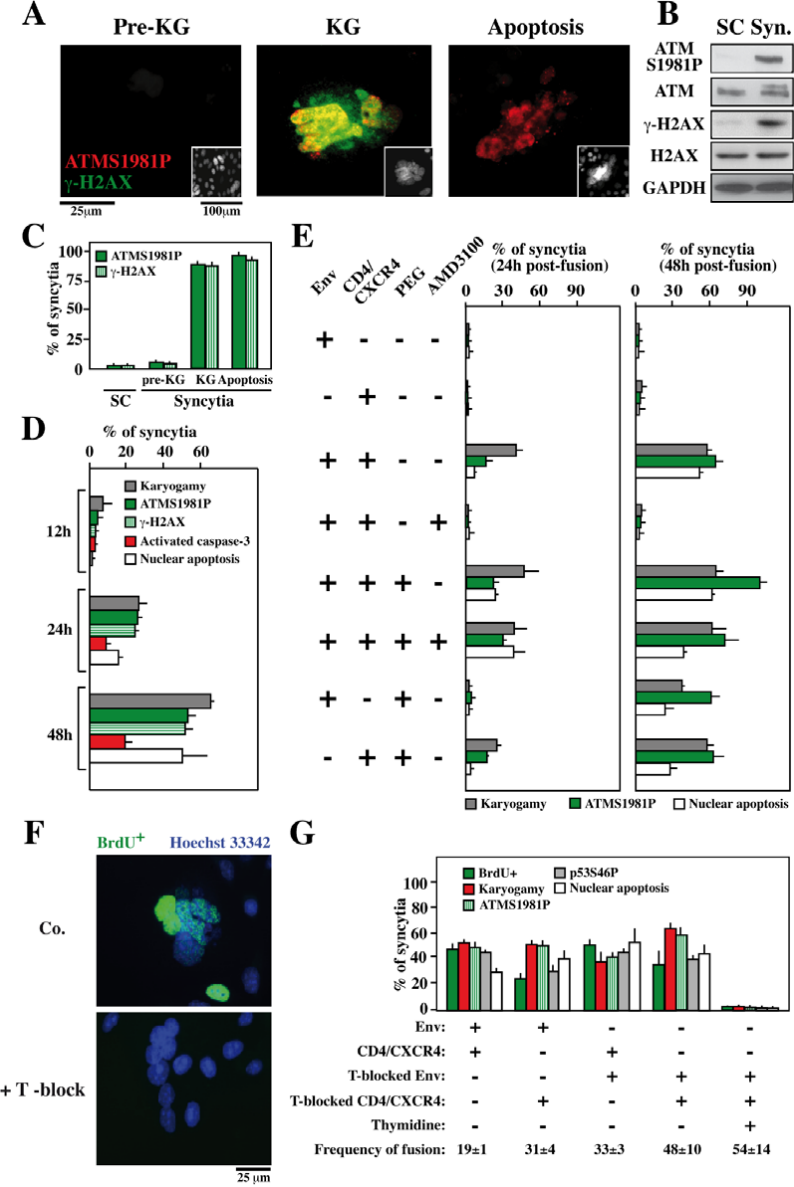
DNA damage foci in karyogamic syncytia elicited by HIV-1 Env. A. ATM and histone H2AX phosphorylation. Syncytia were generated by coculture of HeLa CD4 and HeLa Env cells (36 h), followed by immunofluorescence double staining for the detection of ATMS1981P and γ-H2AX. Representative pre-karyogamic (pre-KG), karyogamic (KG), and apoptotic cells are shown. The inserts depict the staining with the chromatin-specific dye Hoechst 33342. B. Biochemical evidence for ATM and H2AX phosphorylation. Extemporaneous 1∶1 mixtures of HeLa Env and HeLa CD4 single cells (SC) or syncytia (48h, which are mixtures of pre-KG and KG cells) were subjected to immunoblot detection of total ATM, ATMS1981, total H2AX and γ-H2AX. C. Quantitation of ATM and H2AX phosphorylation in distinct categories of syncytia, as determined in A. D. Time course of ATM, H2AX and caspase activation in syncytia, as determined by immunofluorescence stainings with anti-ATMS1981P, anti-γ-H2AX (as in A) and an antibody only recognizing active caspase-3. In addition, karyogamy and apoptotic chromatin condensation was assessed (X±SD, n = 3). E. Role of cell fusion in ATM activation. Single cell (SC) or HeLa Env/HeLa CD4 cocultures were performed in standard conditions (as in A), in the absence or presence of the fusion inhibitor AMD3100. Alternatively, HeLa CD4 and HeLa Env cells were subjected to polyethylene glycol (PEG)-enforced cell fusion, either separatedly or in mixed cultures, maintained in the absence or presence of AMD3100. Twenty four hours and 36 hours later, karyogamy, nuclear apoptosis, ATM phosphorylation was assessed by immunostaining. F,G. Inhibition of ATM activation by cell cycle arrest. Hela CD4 and HeLa Env cells were exposed twice overnight to thymidine (1 mM), which leads to an accumulation (synchronization) of cells in the early S-phase. Then, synchronized (“T-blocked”) or non-synchronized cells were cocultured in the absence or in the presence of thymidine (to leave or maintain the cell cycle blockade). Note that continuous blockade prevents BrdU incorporation (F), karyogamy as well as the phosphorylation of ATM (G). Results are means±SD of three independent experiments, each performed in triplicate.

HIV-1 Env elicits syncytia when cells transfected with HIV-1^LAI^ *Env* are cocultured with cells transfected with *CD4*. In this model, apoptosis occurs only in syncytia, after nuclear fusion (karyogamy) has occurred, affecting ∼50% of syncytia after ∼48 hours [10]. Karyogamic (KG) but not freshly formed (pre-karyogamic) syncytia (pre-KG) nor single cells (SC) exhibited the activating phosphorylation of ATM on serine 1981 (ATMS1981P) within their nuclei (Fig. 1A,C), correlating with the phosphorylation of the quintessential ATM substrate, histone H2AX [31], in discrete γ-H2AX foci (Fig. 1A, C, D). ATM phosphorylation and γ-H2AX foci only affected karyogamic or apoptotic nuclei (Fig. 1E) and occurred in cells in which caspase-3 was not yet activated (Fig. 1D). Importantly, cell fusion was necessary and sufficient for ATM activation, because ATM phosphorylation was abolished in the coculture system by addition of AMD3100 (an inhibitor of Env-dependent fusion), yet could be restored by enforcing cell fusion with polyethylene glycol (PEG), even in the absence of an Env/CD4 interaction (Fig. 1E). Thus any kind of cell fusion, be it mediated by Env or by polythelene glycol, can induce ATM phosphorylation. S-phase blockade by high-dose thymidine completely inhibited both karyogamy and ATM activation, provided that both fusion partners were subjected to cell cycle blockade (Fig. 1F,G). Thus, the activation of DNA damage foci containing ATMS1981P and γ-H2AX is likewise due to cytogamy among non-synchronized cells leading to the fusion of nuclei that are in distinct phases of the cell cycle.

**Figure 2 pone-0002458-g002:**
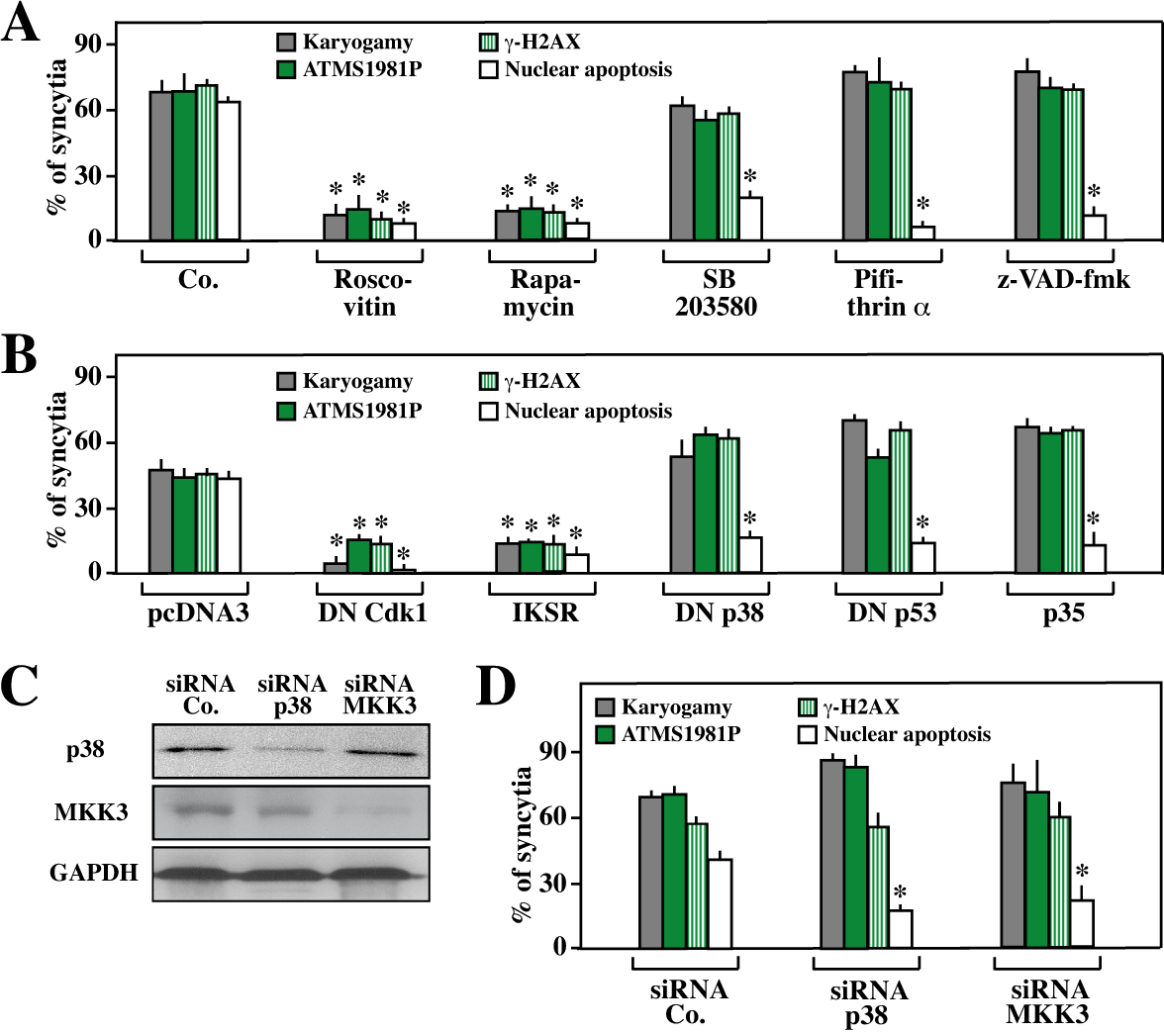
ATM activation occurs after karyogamy but before apoptosis. HeLa CD4 and HeLa Env cells were left untransfected (A) or were transfected with a series of control vectors and dominant-negative (DN) contructs (B) or with specific siRNAs (C, D), cultured separately for 24 h (B) or 36 h (D), and then cocultured for 36 h (A, B, D) in the absence or presence or a series of inhibitors (A) (see Materials and Methods). Finally, the cells were fixed, permeabilized and stained for the detection of karyogamy, apoptosis (nuclear chromatin condensation), ATM or H2AX phosphorylation. The immunoblot shown in C confirms the efficacy of the siRNAs, as measured 36 h after transfection, before coculture. Error bars indicate standard deviations of 4 independent experiments. Asterisks indicate significant (p<0.01, paired Student t test) inhibitory effects as compared to untreated controls (Co. in A), vector-only transfected cells (pcDNA3 in B), or cells treated with a control siRNA (in D).

**Figure 3:**
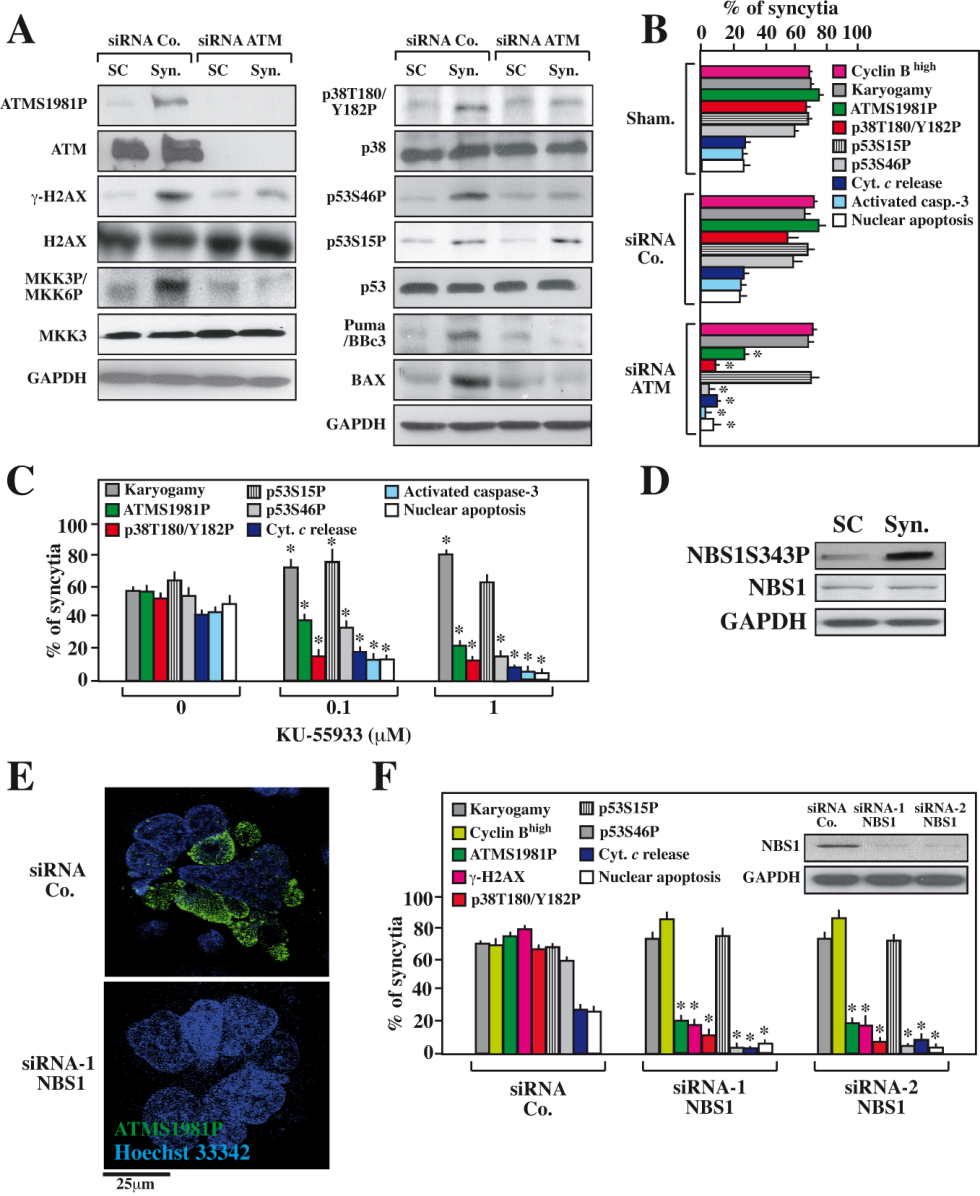
Effect of the ATM knockdown or pharmacological ATM inhibition on the Env-elicited apoptotic signal transduction cascade. HeLa CD4 and HeLa Env cells were separately transfected with the indicated siRNAs and 36 h later the cells were cocultured for further 36 h, followed by the immunoblot detection of the indicated proteins and phoshoproteins (A) or, alternatively, by immunofluorescence detection (B, C) of the indicated parameters (X±SD, n = 5). Alternatively, the cells were not transfected and rather treated with the chemical ATM inhibitor KU-55933 (C), at a dose that did not inhibit karyogamy (100 nM or 1 µM), followed by the determination of the indicated parameters by immunofluorescence analyses (X±SD, n = 3). Moreover, the two cell lines (HeLa CD4 and HeLa Env) were both transfected with a control siRNA or two distinct siRNAs targeting NBS1 (D,E), followed by immunoblot detection of NBS1 after admixture at a 1∶1 ratio (D) or coculture for 36 h and determination of the indicated parameters by immunofluorescence (E, F). Representative fluorescence microphotographs are shown in E and quantitative data (X±SD, n = 3) are summarized in F. Asterisks mark significant inhibitory effects of the ATM-specific siRNA (as compared to a control siRNA in B), of KU-55933 (as compared to untreated cells in C) or of the two NBS1-specific siRNAs (as compared to a control siRNA in F).

**Figure 4:**
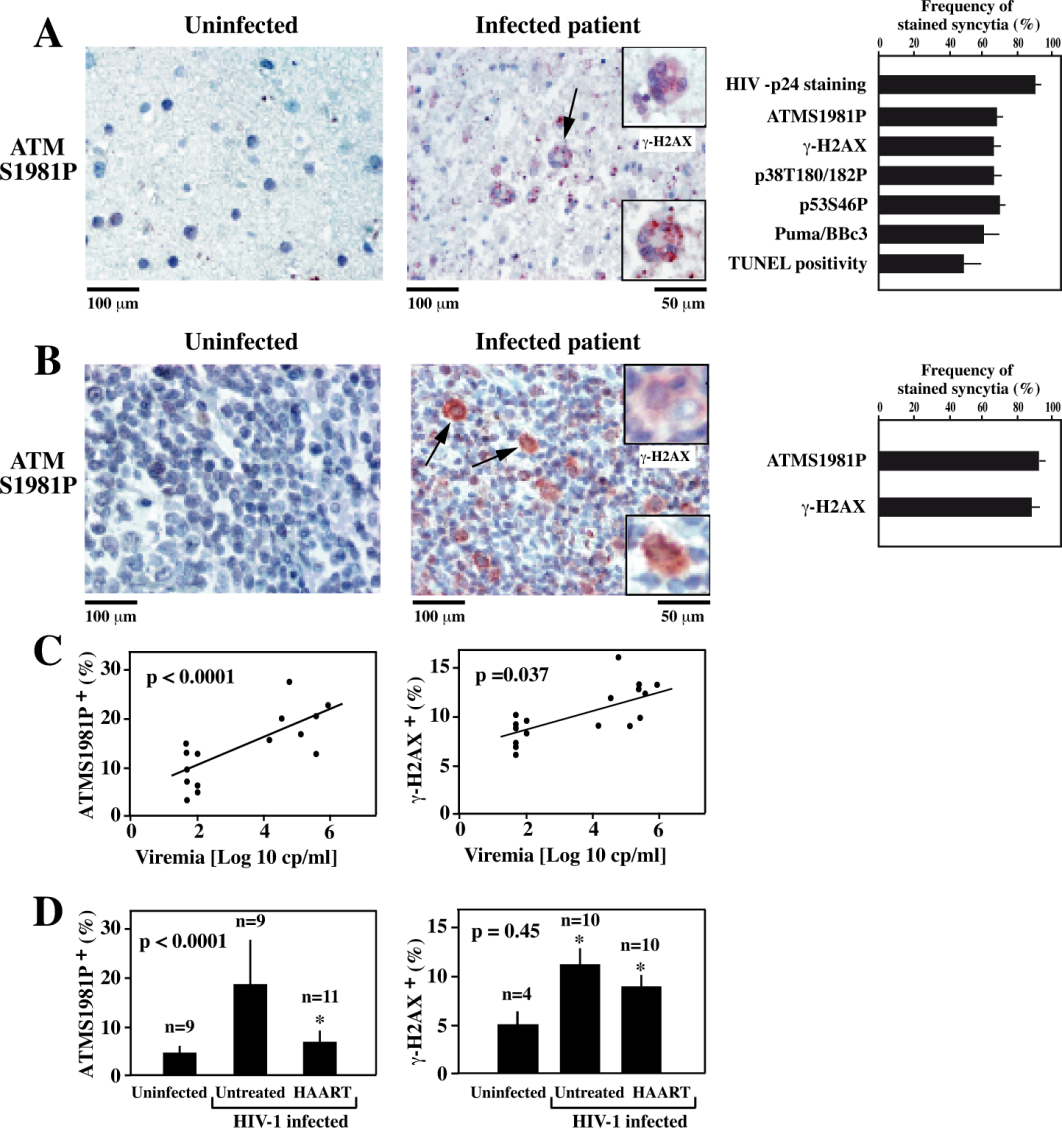
DNA damage foci in vivo, in HIV-1-infected patients. A. Phosphorylation of ATM and H2AX in giant multinuclear cells present in the frontal cortex of patients with HIV-1-associated encephalitis (HAE). Frontal cortex sections from control subjects or untreated HIV-1 carriers without opportunistic infections at the time of death were subjected to the immunohistochemical detection of ATMS1981P or the ATM substrate γ-H2AX (insert), using the same antibodies as in Fig. 1. Arrows indicate syncytia and inserts show higher magnifications. Control brains from HIV-1^−^ subjects did not contain syncytia. The percentage of syncytia (X±SEM) staining positively for the indicated antigens were determined for six different HIV-1 carriers (A). Note that <5% of mononuclear cells were positive for any of the indicated antigens. B. DNA damage foci in lymph nodes from HIV-1 carriers. Lymph node biopsies from control subjects or untreated HIV-1 carriers were stained with the indicated antibodies Arrows and inserts show bona-fide syncytia. C, D. Correlation between ATM activation, H2AX phosphorylation, viral status and HAART treatment. PBMC from untreated patients with distinct levels of viremia were subjected to ATMS1981P and γ-H2AX staining. p values refer to the correlation coefficients (C). In addition, a cohort of uninfected donors, untreated HIV-1 carriers, and HAART-treated subjects with undetectable viral titers were analyzed for ATM activation and γ-H2AX positivity (D). Note that that some but not all of the patient samples could be analyzed for all indicated parameters.

**Figure 5:**
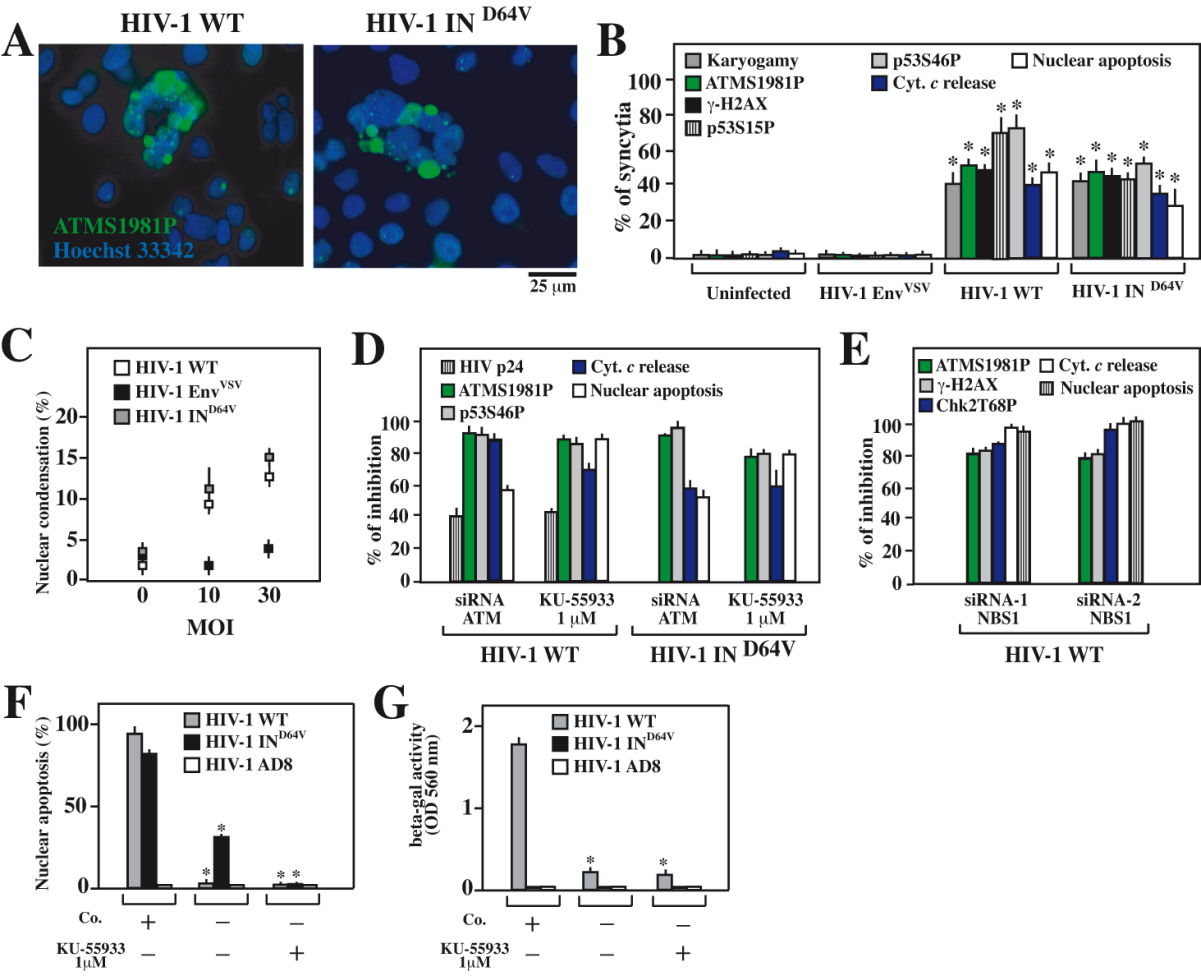
Activation and contribution of ATM to syncytial apoptosis of HIV-1-infected CD4^+^cell lines. A–D. CD4^+^ HeLa cells were infected with HIV-1 WT, HIV-1 Env^VSV^ and HIV1 IN^D64V^ at a MOI of 10 for 38 h and then stained for ATMS1981P (A) or all other markers indicating syncytial apoptosis. The percentages (X±SD, n  = 3) of syncytia exhibiting the indicated characteristics were quantified at an MOI of 10 (B), and the percentage of nuclei with apoptotic chromatin condensation (usual within syncytia) was quantified at different MOI (C). CD4^+^ HeLa cells were either transfected with specific siRNAs depleting ATM or treated with the ATM inhibitor KU-55933. Then, cells were infected with HIV-1 WT or HIV1 IN^D64V^ at a MOI of 10 for 48 h, and the expression of the viral protein p24, the activation of ATM or p53, and apoptosis were quantified (D). Results are plotted as the percent inhibition of the indicated parameters determined for syncytia (excluding single cells) compared to control siRNA transfected or untransfected cells. E. Effect of NBS1 knockdown on syncytial apoptosis of HIV-1 infected CD4^+^ HeLa cells. CD4^+^ HeLa cells were transfected with NBS1-specific or control siRNAs, and infected for 48h with HIV-1 WT (MOI of 10), followed by quantification of the indicated parameters by immunofluorescence. F, G. Effect of ATM inhibition on syncytial apoptosis in HIV-1 infected CEM T cells. Cells were infected with wild type HIV-1 in the presence (or in the absence) of the ATM inhibitor KU-55933, and nuclear apoptosis was determined 72 h later (F). Moreover, the number of infectious viruses contained in the culture supernatant was determined (G). Asterisks in F and G indicate significant inhibitory effects of KU-55933.

**Figure 6:**
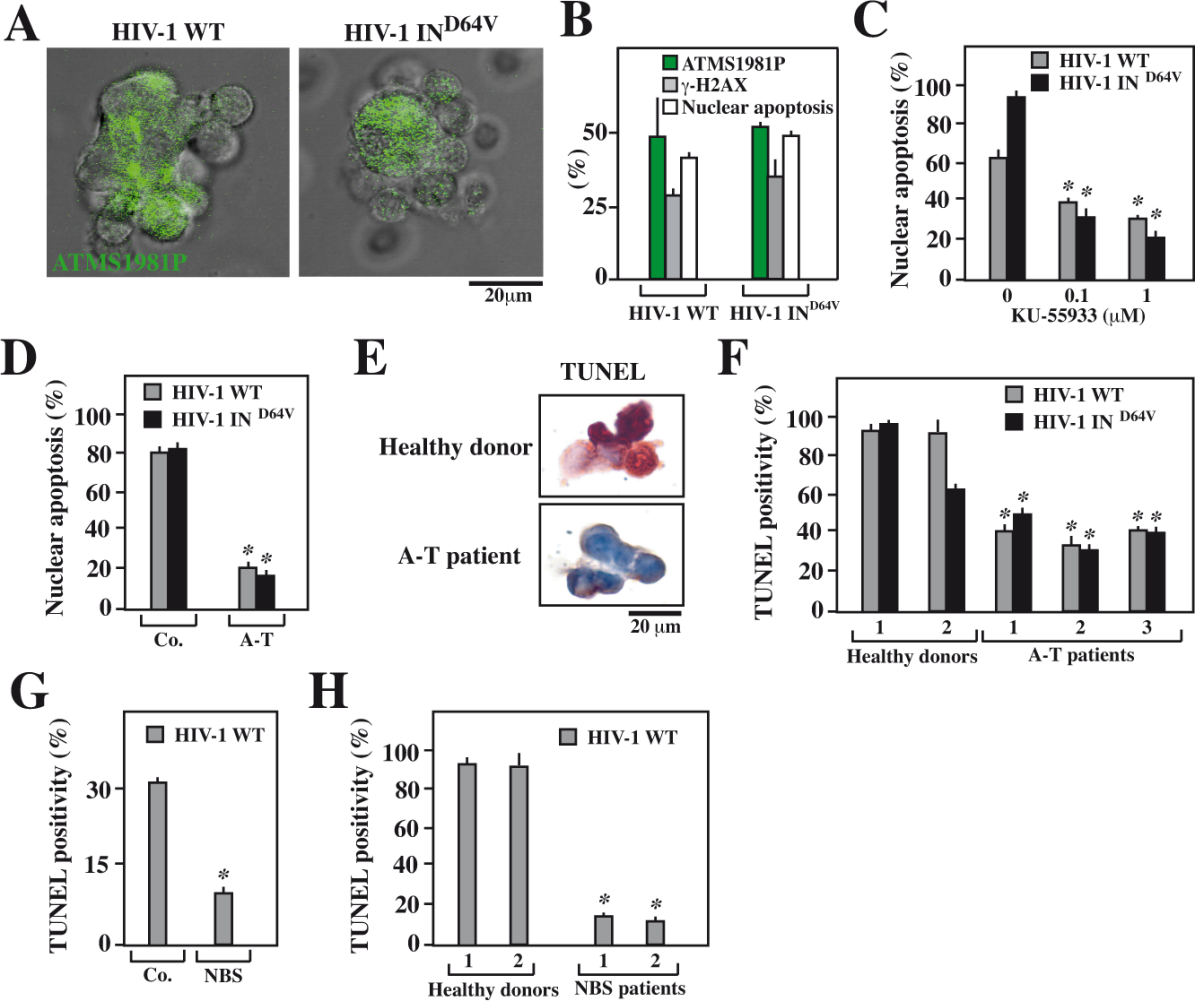
Involvement of ATM and NBS1 in syncytial apoptosis observed in HIV-1 infected human lymphoblasts. PHA/IL-2 lymphoblasts obtained from healthy donors were infected with HIV-1 WT and HIV1 IN^D64V^ at a MOI of 10 for 48 h and then stained for the indicated markers. A. DNA damage foci containing ATMS1981P in syncytia were identified by immunofluorescence after infection of PHA/IL-2 lymphoblasts with HIV-1 WT and HIV1 IN^D64V^. B. Comparative quantification of DNA damage foci induced by infection with HIV-1 WT and HIV1 IN^D64V^ at a MOI of 10 for 48 h. The phosphorylation status of ATM, H2AX and apoptosis were quantified (X±SD, n = 3 experiments). C. Effect of the ATM inhibitor KU-55933 on the apoptosis of HIV-1 infected primary T lymphoblasts. PHA/IL-2 lymphoblasts from healthy donors (n = 3) were infected with the indicated HIV-1 strain (MOI 5, 48 h), followed by Hoechst 33342 staining and quantification of syncytia exhibiting apoptotic chromatin condensation. D. Reduced syncytial apoptosis of Herpes virus saimiri (HVS)-transformed T cells from A-T patients. CD4^+^ T lymphoblasts from an A-T patient and a healthy donor (Co.) were infected for 24 hours with HIV-1 WT or HIV1 IN^D64V^ at a MOI of 10, followed by determination of the frequency of nuclear apoptosis among syncytia by Hoechst 33342 staining. E,F. Comparison of T lymphoblasts from healthy and A-T patients. PHA/IL-2 lymphoblasts from controls or A-T patients (n = 3) were infected by HIV-1 WT, and the frequency of syncytia with Tunel^+^ nuclei was assessed. Representative syncytia are shown in E and quantitative data (X±SD, n = 3) are reported in F. G. Decreased HIV-1-induced apoptosis of immortalized T cells obtained from a patent with Nijmegen breakage syndrome (NBS). Cells from an NBS patient and a healthy donor (Co.) were infected for 48h hours with HIV-1 WT at a MOI of 10, and the frequency of syncytia bearing Tunel-positive nuclei was evaluated. H. Comparison of primary T lymphoblasts from healthy and NBS donors. PHA/IL-2 lymphoblasts from controls (n = 2) or NBS patients (n = 2) were infected. The frequency of syncytial apoptosis was determined by TUNEL assay. Asterisks indicate significant (p<0.01) effects of KU-55933, NBS or A-T-mutations as compared to the respective untreated or unmutated control cells.

**Figure 7:**
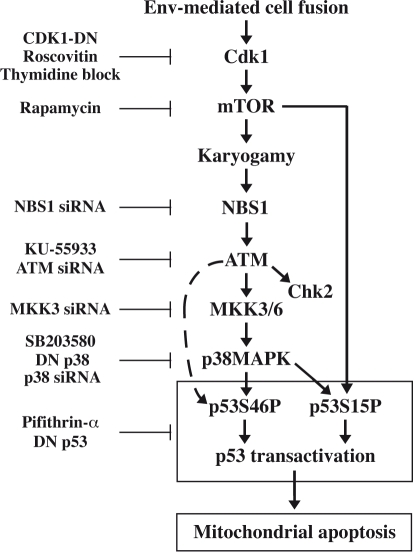
Hypothetical signaling cascade leading to syncytial apoptosis. Sequential events leading to syncytial cell death in HIV-1 infection are shown. All inhibitory agents used throughout the paper are listed.

When karyogamy was inhibited by roscovitin (a chemical inhibitor of Cdk1), a dominant-negative (DN) mutant of Cdk1, rapamycin (an inhibitor of mTOR), or transfection with the inhibitor of NF-κB super-repressor (IKSR), ATM and H2AX phosphorylation were inhibited (Fig. 2A,B) underscoring the fact that DNA damage foci are only formed as a result of karyogamy. Inhibition of p38 MAPK (with SB203580, transfection with DN p38, or small interfering RNAs [siRNAs] specific for p38 (or for its obligate activator MAPK kinase 3 [MKK3], Fig. 2C), p53 (with cyclic pifithrin-α or DN p53) or caspases (with *N*-benzyloxycarbonyl-Val-Ala-Asp-fluoromethylketone [Z-VAD-fmk] or the baculovirus inhibitor of apoptosis protein p35), all suppressed apoptosis as an internal control of their efficacy, yet had no effect on ATM phosphorylation (Fig. 2A–D). Thus, DNA damage foci are formed independently from p38, p53 and caspase activation. Altogether, these findings indicate that ATM becomes activated as a result of cellular and nuclear fusion, upstream of the p38/p53-dependent apoptotic cascade.

### ATM and its activator NBS1 are required for the activation of p38 MAPK and p53

Driven by the finding that ATM is activated in Env-elicited syncytia, we determined the contribution of ATM to the induction of syncytial apoptosis. Inhibition of ATM by a specific siRNA [32] (Fig. 3A,B) or by the chemical inhibitor KU-55933 [33] (Fig. 3C) resulted in the expected inhibition of H2AX phosphorylation (Fig. 3A), indicating that H2AX phosphorylation was indeed mediated by ATM, exactly as this occurs in “classical” DNA damage foci. ATM inhibition did not prevent the fusion of cells or nuclei (karyogamy) (Fig. 3B,C) and KU-55933 actually tended to increase the frequency of karyogamic syncytia (Fig. 3C), presumably because it inhibited their apoptotic demise. Indeed, both the knockdown and the pharmacological inhibition of ATM resulted in an inhibition of syncytial apoptosis, as indicated by a decrease in cytochrome *c* release from mitochondria, a reduction of caspase-3 activation, and a decline in nuclear apoptosis. ATM depletion or KU-55933 reduced upstream signaling events that stimulate syncytial apoptosis such as the activating phosphorylation of p38 MAPK at T180/Y182 (p38 T180/Y182P). Accordingly, ATM inhibition reduced the phosphorylation of p53 at S46 (p53S46P), which is mediated by p38 MAPK [20]. However, ATM knockdown or inhibition did not affect the phosphorylation of p53 at S15 (p53S15P) that is closely linked to karyogamy [25] (Fig. 3). The DNA damage-induced activation of ATM depends on the protein Nijmegen breakage syndrome 1 (NBS1) [34]–[38]. This also applies to syncytia because the siRNA-mediated depletion of NBS1 (Fig. 3D) strongly inhibited the phosphorylation of ATM and H2AX (Fig. 3E,F), correlating with a reduction of p38 T180/Y182P, p53S46P and apoptosis. However, NBS1 depletion exerted no inhibitory effects on karyogamy and p53S15P (Fig. 3F), exactly as was observed for ATM depletion (Fig. 3A,B). Altogether, these results unravel a hierarchy of molecular events in which ATM, as well as its obligate activator NBS1, regulate the MAPK cascade leading to the phosphorylation of p53 on S46 in HIV-1 Env-induced syncytia.

### HIV-induced ATM phosphorylation in vivo

HIV-1-induced syncytia are most easily discernible in histological sections of the frontal cortex, where they constitute a pathognonomic sign of HAE [16], [17]. We found that the nuclei of such HAE-associated multinuclear giant cells exhibited ATM phosphorylation and γ-H2AX foci, while normal neurons or glial cells were mostly negative for these markers, as determined by immunohistochemistry (Fig. 4A). The frequency of brain syncytia with phosphorylated ATM was higher than that of syncytia exhibiting DNA fragmentation (as determined by terminal desoxy-uridine nucleotide end labeling [TUNEL] staining) (Fig. 4A), thus corroborating the notion that ATM activation occurs before apoptosis. In lymph nodes from untreated HIV-1 carriers, bona fide syncytia and a fraction of mononuclear cells exhibited an enhanced nuclear staining for ATMS1981P and γ-H2AX (Fig. 4B). Similarly, a fraction of peripheral blood mononuclear cells (PBMC) from untreated HIV-1^+^ donors contained activated ATM and γ-H2AX, correlating with viral load (Fig. 4C). The frequency of PBMC with phosphorylated ATM dropped upon successful HAART (Fig. 4D), although γ-H2AX remained high, perhaps due to the DNA-damaging effects of HAART [39]. Altogether, these data indicate that the activation of ATM also occurs in vivo, in the brain, lymph nodes and circulating immune cells of patients with HIV-1 infection.

### Requirement of NBS1 and ATM for apoptosis induction by HIV-1

Within 48 hour after infection of CD4^+^CXCR4^+^ HeLa cells with the syncytium-inducing HIV-1^LAI^ strain, a significant fraction of cells, preferentially syncytia, exhibited mitochondrial cytochrome *c* release, caspase-3 activation and apoptotic chromatin condensation (Fig. 5A,B). An HIV-1 mutant lacking integrase activity due to a point mutation (HIV-1 IN^D64V^) but otherwise identical to the wild type (WT) HIV-1^LAI^ strain, induced a similar degree of syncytial apoptosis. In contrast, much less syncytium formation and apoptosis were observed when the endogenous HIV-1 *env* gene was replaced by that of VSV (HIV-1 Env^VSV^) (Fig. 5B,C), confirming the cardinal role of Env in short-term apoptosis induction by HIV-1 [8], [9]. Importantly, infection with HIV-1 IN^D64V^ (but not HIV-1 Env^VSV^) was as efficient in eliciting ATM phosphorylation as HIV-1^LAI^, both in CD4^+^CXCR4^+^ HeLa cells (Fig. 5B) and in CEM cells (not shown).

siRNA-mediated ATM knockdown or pharmacological ATM inhibition with KU-55933 strongly inhibited (by >70%) apoptosis induction by HIV-1 WT or HIV-1 IN^D64V^ in HeLa CD4^+^ cells (Fig. 5D). In addition, depletion or inhibition of ATM partially reduced (by 30–40%) the expression of p24 by HIV-1^LAI^–infected cells (which is undetectable in HIV-1 IN^D64V^ infected cells) (Fig. 5D), thus corroborating previous studies showing that ATM can influence viral replication [33]. Knockdown of NBS1 also reduced viral replication and HIV-1 induced apoptosis, upstream of ATM (Fig. 5E). The depletion/inhibition of ATM (or the depletion of the ATM activator NBS1) blocked the p38 MAPK/p53S46P pathway that leads to apoptosis in HIV-1 WT or HIV-1 IN^D64V^-induced syncytia (Fig. 5D,E). KU-55933 also reduced the cytopathic effect of HIV-1 WT or HIV-1 IN^D64V^ on CEM T lymphoma cells (Fig. 5F) and partially decreased the replication of HIV-WT in these cells (Fig. 5G).

Primary PHA blasts infected by HIV-1 in vitro manifested ATM phosphorylation (ATMS1981P) within syncytia (Fig. 6A). This result was obtained after infection with either HIV-1 WT and HIV-1 IN^D64V^, corroborating the fact that retroviral integration into the host genome (which depends on the HIV-1 integrase) is not required for activation of the ATM. Indeed the frequency of syncytia exhibiting the phosphorylation of ATM and that of the ATM substrate γ-H2AX was similar after infection with HIV-1 WT and HIV-1 IN^D64V^ (Fig. 6B). Moreover, the ATM inhibitor KU-55933 suppressed syncytial apoptosis of primary T lymphoblasts infected with HIV-1 WT or HIV-1 IN^D64V^ with a similar efficiency (Fig. 6C). To provide a genetic proof that ATM is required for syncytial apoptosis induced by HIV, we took advantage of HSV-transformed T lymphoblasts from patients with ataxia telangiectasia (A-T), which results from a loss-of-function mutation of ATM. Such transformed A-T lymhoblasts formed syncytia in response to infection by HIV-1^LAI^ or HIV-1 IN^D64V^. A-T syncytia underwent less apoptosis than control syncytia obtained by infection of T HSV-transformed lymphoblasts from a healthy donor (Fig. 6D). Moreover, primary T lymphoblasts from patients with A-T exhibited less syncytial apoptosis upon infection by HIV-1 WT or HIV-1 IN^D64V^ than cells from healthy donors (Fig. 6E,F). Spontaneously immortalized T lymphoblasts derived from NBS patients also formed syncytia after HIV-1 infection that were less prone to apoptosis than cells from control subjects (Fig. 6G). Finally, when HIV-1 elicited syncytia among primary T lymphoblasts from NBS patients, these syncytia failed to undergo apoptosis in conditions in which near-to-all syncytia from healthy control patients succumbed to apoptosis (Fig. 6H).

Altogether, these results unravel the critical role of ATM and NBS1 in syncytial apoptosis of primary T lymphocytes infected by HIV-1.

## Discussion

The search for host genes that are required for HIV-1 replication and HIV-1-mediated cytopathic effects is expected to furnish new pharmacological targets for HIV-1 therapy that, in contrast to virus-encoded targets, are near-to-invariant and far less prone to mutation. As shown here, syncytium formation induced by Env (in an HIV-1-free system, Fig. 1–3), wild type HIV-1 (Fig. 5–6), or integrase-defective HIV-1 (Fig. 5–6) triggers an apoptotic pathway that involves the activation and action of ATM. Previous reports suggested that ATM is involved in the full chromosomal integration of the HIV-1 genome [33], [40]. The present study delineates a novel role of ATM in HIV-1 infection, in a pro-apoptotic signal transduction pathway that is independent of HIV-1 integrase, but dependent on the fusogenic action of Env (Fig. 1, 5–6). Our results indicate that in Env-elicited syncytia, the activating ATM phosphorylation is an early event that is triggered by karyogamy. As a possibility, ATM (and its upstream activator NBS1) might sense incomplete DNA replication by one of non-synchronized nuclei as DNA damage and/or become activated by cellular stress elicited by syncytium formation [41].

ATM is required for stimulating the MAPK pathway (MKK3→p38MAPK) that culminates in p53 phosphorylation on serine 46 (Fig. 2). Reportedly, ATM may mediate the direct phosphorylation of p53 on serine 46 [42]. However, we have no direct evidence that this occurs in Env-elicited syncytia, and we prefer in our current working hypothesis that ATM stimulates p53S46P in an indirect fashion through the activation of p38MAPK (see below, Fig. 7). All the components of this pathway (ATM→p38MAPK→p53) have been detected in vivo, in multinuclear giant cells of the HAE brain, lymph node biopsies and a fraction of PBMC (see Fig. 4 as well as references [19]–[21], [24], [43]). Thus, the data obtained in vitro, in the syncytial model of apoptosis elicited by Env can be extrapolated, at least to some degree, to AIDS pathogenesis.

There are multiple mechanisms through which ATM can modulate p53 activity. Certainly, ATM depletion (or that of its activator NBS1) does not influence the cell cycle of undamaged single cells or of syncytia (with absent effects on the frequency of cyclin B1-positive cells, and on the G2/M-linked karyogamy, Fig. 3). Although recombinant ATM phosphorylates p53 on serine 15 in vitro [44], [45], inhibition of ATM did not affect p53S15P phosphorylation in HIV-1 Env-elicited syncytia (Fig. 3). Moreover, it appears that ATM influences p53S46 phosphorylation in an indirect fashion, through the MKK3/p38 MAPK pathway. Accordingly, chemical or genetic inhibition of ATM prevented the activation of MKK3 and p38 MAPK (Fig. 3), which is the principal p53S46 kinase operative in HIV-1 elicited syncytia [20]. ATM is also required for stress-induced p38 MAPK activation after γ-irradiation [46], [47], suggesting that p38 MAPK may constitute an indirect molecular link between ATM and p53 activation in various systems, not only in syncytial apoptosis.

Here we show for the first time that HIV-1 infection is accompanied by the formation of “DNA damage foci” in patient's tissues. Such DNA damage foci are typically detected by antibodies that recognize γ-H2AX. The phosphorylation of H2AX can be mediated by ATM [31] as well as by ATR [48], and ATR reportedly can be activated by retroviral DNA integration [49] as well as by the HIV-1 gene product Vpr [50]. Vpr suffices to elicit γ-H2AX foci when introduced into cells, for instance by transduction with a Vpr-expressing lentivirus [51]. However, Env is sufficient to induce γ-H2AX foci in a virus-free system (Fig. 1–3), meaning that γ-H2AX foci can occur in the absence of Vpr. Moreover, knockdown of ATR did not inhibit γ-H2AX foci in our system (not shown), while inhibition of ATM fully blocked H2AX phosphorylation (Fig. 3). It should be noted that, as ATR, ATM has previously been reported to be activated by HIV-1, through the integration of HIV-1 into the host genome [33]. However, in our system an integrase-deficient HIV-1 was able to induce γ-H2AX foci within syncytia (Fig. 5), and syncytia induced by Env in a virus-free system also manifested γ-H2AX foci in an ATM-dependent fashion (Fig. 1–3). Thus, syncytium formation triggered by Env can induce a DNA damage response in the absence of proviral integration. In tissue sections from HIV-1-infected patients, more than 60% of multinuclear giant cells (syncytia) stained positively for γ-H2AX foci (Fig. 4), suggesting that syncytium formation is indeed linked to the activation of a disease-relevant DNA damage response.

Kinases are readily amenable to specific pharmacological inhibition. At least three kinases of the phosphatidylinositol 3-kinase family, namely ATM, mTOR and DNA-dependent protein kinase (DNA-PK), operate at two different levels of AIDS pathogenesis. At a first level, mTOR [25] and ATM (this paper) act as pro-apoptotic signal transducers and are required for Env-dependent cell death induction. At a second level, ATM [33], [40] and DNA-PK [52] facilitate retroviral integration into the host genome, and their inhibition can provoke the apoptotic abortion of the host cells [33], [52]. Thus, ATM inhibition might have a dual beneficial effect on HIV-1 infection, by inhibiting Env-elicited bystander killing and by triggering the apoptotic destruction of freshly infected cells. Although long-term pharmacological inhibition of ATM might have deleterious oncogenic side effects, the therapeutic effect of short-term inhibition of ATM should be evaluated in AIDS.

## Materials and Methods

### Antibodies, plasmids and reagents

Polyclonal rabbit antibodies from Cell Signaling Technology were used for detection of Chk2, Chk2T68P, MKK3, MKK3 with phosphorylated serine 189 and/or MKK6 with phosphorylated serine 207 [MKK3/6 S189/207P], p38, p38T180/Y182P, p53S15P, and p53S46P. The monoclonal anti-cyclin B1, anti-cytochrome *c* were obtained from Becton Dickinson. The polyclonal rabbit antibody against NBS1 was obtained from Calbiochem. The monoclonal antibody used for detection of NBS1S343P was from Interchim. Monoclonal antibodies against ATM, ATMS1981P, H2AX H2AXS139P, hMRE11 and Rad50 were purchased from Upstate and anti-GFP used from Invitrogene. The plasmid for Baculovirus p35 was a kind gift from Guy Salvesen (Burnham Institute, La Jolla, CA). DN mutant plasmids for cyclin-dependent kinase (DN Cdk1), inhibitor of NF-kappaB superrepressor (IKSR), DN p53 (H175), DN p38 MAPK were previously described [19]. For expression of human immunodeficiency virus type 1 (HIV-1) in cell lines, the proviral clone pLN4-3 was used as the template. Molecular clone pLN4-3 containing the inactivating integrase mutation Q62A (HIV-1 IN-) was a gift from C. Petit and P. Sonigo (Institut Pasteur, Paris, France). Molecular clone pLN4-3 deleted in envelope gene (HIV-1 ENV-) was a gift from O. Schwartz (Institut Pasteur). The Cdk1 inhibitor Roscovitin and p38MAPK inhibitor SB203580 were from Calbiochem-Novabiochem. AMD3100, mTOR inhibitor rapamycin and polyethylene glycol (PEG) were from Sigma-Aldrich. p53 inhibitor cyclic pifithrin-α and the pan-caspase inhibitor N-benzyloxycarbonyl-Val-Ala-Asp-fluoromethylketone, ZVAD.fmk were from Bachem. The pharmacological ATM inhibitor KU-55933 was obtained from Dr. Mark O'Connor and Graeme Smith (Kudos, Cambridge, UK).

### Cell lines, cell culture, transfection and RNA interference

HeLa cells stably transfected with the Env gene of HIV-1 LAI/IIIB(HeLa Env) and HeLa cells transfected with CD4 (HeLa CD4) were cultured at a 1∶1 ratio in Dulbecco's modified Eagle's medium supplemented with 10% FCS, 2 mM L-glutamine and penicillin/streptomycin (Invitrogen) in the absence or in presence of 1 µM roscovitin, 1 µM rapamycin, 100 nM SB203580, 10 µM cyclic pifithrin-α or 100 µM ZVAD.fmk during 36 hours. Human lymphoid cell line CEM4fx was grown in RPMI medium (Gibco). Transfections of plasmids were performed with lipofectamine 2000 (Invitrogen) 24 hours before cell fusion. Only experiments in which transfection of a GFP indicator gene affected >60% of single cells were included in the analysis. For RNA interference, we used published siRNAs specific for ATM [32], NBS1 (hNBS1.1 CAGGAGGAAGATGTCAATG dTdT or hNBS1.2 GGCGUGUCAGUUGAUGAAA dTdT), MKK3 (hMEKK3-258 GCACGGUCGACUGUUUCUA dTdT), p38MAPK (Dharmacon RNATtechnology) that were transfected into cells with Oligofectamine (Invitrogen). Herpesvirus saimiri-immortalized CD4^+^ T cells from a patient with A-T (ATM mutation 5908 C>T) and from a control donor [53], as well as spontaneously immortalized T cells from NBS patient (NBS mutation 657de15) and for a control donor were cultured as described [54].

### Immunoblots, immunofluorescence and immunohistochemistry

Whole cell protein lysates were prepared by resuspending cells in 250 mM NaCl-containing lysis buffer (250 mM NaCl, 0,1% NP40, 5 mM EDTA 10 mM Na_3_VO_4_, 10 mM NaF, 5 mM DTT, 3 mM Na_4_P_2_O_7_ and protease inhibitor cocktail). After brief sonication and centrifugation, proteins quantification was performed according to standard procedures. Proteins (60 µg) were separated by 3% or 15% SDS-PAGE, transferred to nitrocellulose membrane at 4°C overnight, blocked in 10% milk for 1h buffer, incubated during 1h30 with the first antibody at room temperature and 1h with the second appropriate antibody (goat anti-rabbit or goat anti-mouse; Southern Biotechnology) conjugated to horse radish peroxidase and revealed with the enhanced ECL chemiluminescence detection system. For phosphorylation detection, saturation and antibodies incubation were performed in the blocking buffer (Zymed) during 10 hours. To identify proteins attached to damaged nuclear matrix by immunofluorescence microscopy, living cells were incubated during 20 minutes, 10 minutes and 5 minutes with a fractionation buffer (containing 50 mM Hepes, 150 mM NaCl, 1 mM EDTA, 0,2% NP40, 10 mM NaF, 10 mM β-glycerophosphate, 1 mM Na_3_VO_4_ and protease inhibitor cocktail) at 4°C before fixation. Then, immunofluorescence and immunohistochemistry were performed following published procedures [20], [21].

### Viral and pseudo-viral constructs

To produce viral stocks, 293T cells were transfected with virus-encoding plasmids by the calcium phosphate method Briefly, 293T cells (2.5×10^5^) were plated on 75 cm2 (T-75) culture flasks and transfected the following day either with 12 µg of pNL4-3 HIV-1, INQ62ApNL4-3 or ΔEnv pNL43 for wild type HIV-1, HIV-1 IN- and HIV-1 ENV- viral stock respectively. For VSV G-pseudotyped HIV-1 ENV- viral stock, 293T cells were cotransfected with 12 µg of ΔEnv pNL43 plasmid and 5 µg of pMD.G plasmid. Following the incubation period (12 h), the transfection mixture was replaced with 10 ml of fresh growth medium. Then, 24 h later, the media containing the first batch of virus was harvested and 10 ml of fresh growth medium was added to the cells. Upon collection, all virus-containing media was low-speed centrifuged, filtered through a 0.45-µm low-protein-binding Durapore filter (Millipore) to remove cell debris, and stored in 1-ml aliquots at −80°C. Viral stocks were standardized by means of the HIV-1 P24 ELISA assay (PerkinElmer) and infection of P4 cells followed by cell fixation and X-Gal staining as previously described. Pseudotyped viruses were produced by a recombinant virus assay. Briefly, 293T cells were cotransfected with a plasmid encoding Env NL43 and a plasmid encoding the viral envelope of vesicular stomatitis virus G (VSV-G). Cells were grown for 72 h, and the resulting viruses were used to infect P4 cells in the presence of increasing drug concentrations.

### Patients

Axillary lymph node biopsies were obtained from healthy and HIV-1-infected individuals (all males, mean age 36 years, with a plasma viral load >10^5^ copies/ml) after informed consent. Plasma HIV-1 RNA levels were determined by the bDNA procedure (Versant HIV RNA 3.0; Bayer, USA). Post mortem frontal cortex sections were obtained from 6 brains of patients with HIV-1 associated dementia (but lacking secondary infections) and 3 control brains obtained from uninfected control patients. Three patients with ATM mutations and clinical signs of A-T (patient 1: male, 18 years, heterozygote for two truncating ATM mutations, 7792C>T and 8283delTC, patients 2 and 3: two sisters of 30 and 28 years, homozygotes for ATM mutation 9139C>T), two patients with NBS1 mutations and clinical signs of NBS were enrolled in this study and compared to age- and sex-matched healthy controls. Human samples were obtained with written informed consent in accord with the National and European legal requirements, after approval by the Institutional Review Board of the National Institute for Infectious Disease and the Medical Faculty of the University of Rome.
